# Retracted: MicroRNA‐224 promotes the sensitivity of osteosarcoma cells to cisplatin by targeting Rac1

**DOI:** 10.1111/jcmm.14431

**Published:** 2019-07-24

Retraction: Chenglin Yang, MicroRNA‐224 promotes the sensitivity of osteosarcoma cells to cisplatin by targeting Rac1 2016, Vol 20, Issue 9 (https://doi.org/10.1111/jcmm.12852). The above article, published online on 25 May 2016 in Wiley Online Library (wileyonlinelibrary.com), has been retracted by agreement between the authors, the journal Editor in Chief Stefan Constantinescu, and John Wiley & Sons Ltd. The retraction has been agreed due to proper consent not having been received from all of the subjects in the study.

